# Supplementary material for: Identification of the Primary Factors Determining the Specificity of Human VKORC1 Recognition by Thioredoxin-Fold Proteins
Source: Int J Mol Sci. 2021 Jan 14;22(2):802. doi: 10.3390/ijms22020802 (PMC7835823; doi:10.3390/ijms22020802)
Supplement: Supplementary file 1 [file ijms-22-00802-s001.pdf]

Supplementary Material (S)

Identification of the Primary Factors Determined the Specificity of VKOR Recognition by Thioredoxin-fold Proteins

Maxim Stolyarchuk, Julie Ledoux, Elodie Maignant, Alain Trouvé and Luba Tchertanov

CONTENT

Tables: Table S1 – Table S4

Figures : Figure S1 - Figure S14

Table S1. Structures of the thioredoxin-fold proteins (from human) deposited in Protein Database PDB [1]. The PDB identification code, method, protein domain studied, protein state, active site motif and reference are presented. Structures used in this study are highlighted in blue.

Protein	PDB code	Method/ Resolution,	Domain	State	CX_1X_2C Motif	Reference
PDI	1x5c	NMR	**a'**	reduced	CGHC	To be published
PDI	1mek	NMR	**a**	oxidized	CGHC	[2]
PDI	3uem	X-ray, 2.29 Å	**bb'a'**	reduced	CGHC	[3]
PDI	4ekz	X-ray, 2.51 Å	**abb'xa'**	reduced	CGHC	[4]
PDI	4el1	X-ray, 2.88 Å	**abb'xa'**	oxidized	CGHC CGHC	[4]
PDI	6i7s	X-ray, 2.50 Å	**abb'xa'**	reduced	CGHC CGHC	[5]
ERp18	1sen	X-ray, 1.20 Å	**a**	oxidized	CGAC	To be published
ERp18	2k8v	NMR	**a**	oxidized	CGAC	[6]
Tmx1	1x5e	NMR	**a**	reduced	CPAC	To be published

Table S2. Sequence identity / similarity in ERp18, PDI, Tmx1 and Tmx4. The sequences of domain **a** from the experimentally determined structures of ERp18, PDI, and Tmx1, as well as the 129 amino acid fragment from the Q9H1E5 sequence of Tmx4 (https://www.uniprot.org/uniprot/) were aligned with ERp18, that was used as a reference for alignment and numbering. The identity / similarity matrix (in%) was calculated using the Needle program from EMBOSS [7] after aligning the sequences of domain **a** (full-length), fragments F1 (33-50) and F2 (67-84).

Full-length	**ERp18**	**PDI**	**Tmx1**	**Tmx4**
**ERp18**	100/100	23/38	15/23	15/23
**PDI**	23/38	100/100	26/42	23/38
**Tmx1**	15/23	26/42	100/100	47/68
**Tmx4**	15/23	23/38	47/68	100/100
F1(17-aas); ***F2***(17-aas)				
**ERp18**	100/100	22/22	11/22	11/17
**PDI**	60/70	100 /100	0/0	0/0
**Tmx1**	40/60	60/70	100/100	50/67
**Tmx4**	30/60	60/70	80/90	100/100

Table S3. L-loop of hVKORC1: Residues involved in non-covalent intramolecular interactions and those available for intermolecular interactions

Interactions and parameters :

- H-bond criteria:
  - Distance D-A < 3.6 Å; Angle at H (DHA) > 120
  - Considered donor/acceptor (D/A) atoms: N, O, S
  - Contacts stabilising the secondary structure (helix) are not considered
- Hydrophobic contacts:
  - Residues: F, A, M, I, L, V, P, F, G
  - Distance < 4 Å

Color code: **positively charged – negatively charged – polar not charged -hydrophobic**

INTRAMOLECULAT INTERACTIONS

**Object of study**	**H-bond**	**Hydrophobic Contact**
The 'closed' conformations, clusters C1^{m}-C5^{m}	**D36, D38, R40, D44, R53, R61, E67**	**A41, A48, G46, I49, V54, L70, L76**
The 'open' conformations, clusters C6^{m}	**R35, D36, R37, D38, Y39, S50, R53, S56, S57, R58, W59, R61, N77**	**V45, F55, F63, L70, L76**

AVAILABILITY TO INTER-MOLECULAR INTERACTION

**Object of study**	**H-bond**	**Hydrophobic Contact**
The 'closed' conformations, clusters C1^{m}-C5^{m}	**R33, R35, R37, Y42, R43, C46, T50, C51, S52, S56, S57, R58, W59, H68, D73, N77**	**A34, L42, V45, F55, F63, L65, V66, V69, L70, L76**
The 'open' conformations, clusters C6^{m}	**D36, E67**	**G56, I49**

Table S4. Helical fold (top) of L-loop in isolated hVKORC1 (**A**), in **Model 1** and **Model 2** (**B**); (bottom) of the PDI in Model 1 and Model 2 (**C**).

A. Isolated L-loop

**Cluster**	**Helical folding, %**
1	29
2	27
3	34
4	25
5	34
6	20

B. L-loop of hVKOR (complex PDI-hVKOR1C)

**Conformation**	**Helical folding, %**
**Model** 1	
t = 10 ns	25
t = 60 ns	36
t = 80 ns	38
**Model 2**	
t = 10 ns	25
t = 60 ns	31
t = 80 ns	15

C. The PDI folding in PDI-hVKOR1C complex

**Conformation**	**Folding proportion, % (total / helix / sheet)**
PDB 4ekz, domain a	60 / 39 / 21
**Model 1**	
t = 10 ns	56 / 33 / 23
t = 60 ns	57 / 37 / 20
t = 80 ns	58 / 38 / 20
**Model 2**	
t = 10 ns	60 / 39 / 21
t = 60 ns	56 / 37 / 22
t = 80 ns	59 / 38 / 21

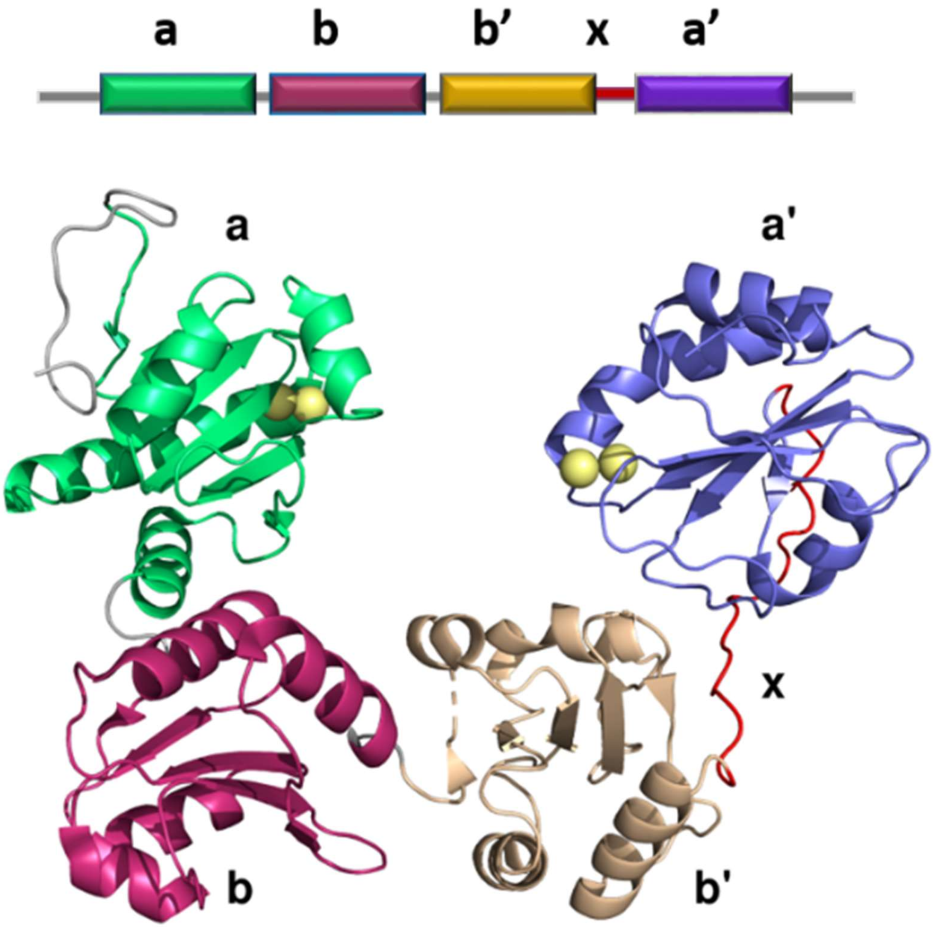

Figure S1. Crystallographic structure of PDI. (Top) Schematic representation of the multidomain PDI. (Bottom) Structure of PDI in the reduced state (PDB ID: 4ekz) is shown as ribbons with the domains distinguished by color identical to the scheme at the top – **a** is in green, **à** is in violet, **b** is in dark red and **b′** is in brown. The cysteine residues from the CGHC motif are shown as yellow balls. The **b′** and **a′** domains are connected with inter-domain linker **x** (in dark red). The domain **a** was used in this study.

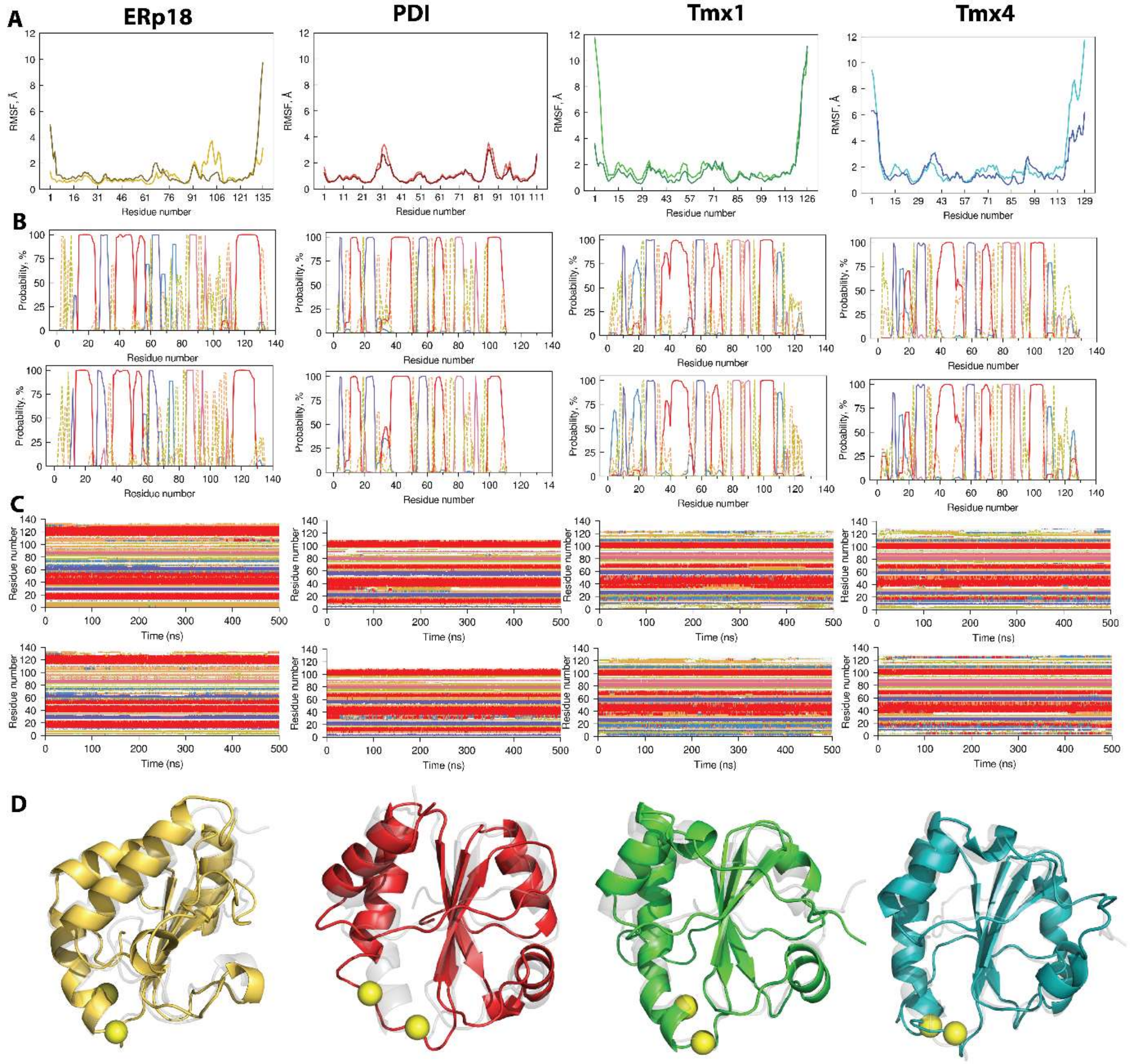

Figure S2. MD simulations of ERp18, PDI, Tmx1 and Tmx4. (**A**) RMSFs computed on the all Cα-atoms for two replica of MD simulations of each protein after fitting on initial conformation. (**B**) Proportion (given as a probability) of every secondary structure type for each residue, as assigned by DSSP. Assignment of the secondary structure type to colors is given as follows: α-helix is in red, 3_{10}-helix is in blue, the parallel and antiparallel strands are in green and violet respectively; turn is in orange and bend is in dark yellow. (**C**) The time-depended evolution of the secondary structure of each residue as assigned by DSSP: α-helix is in red, 3_{10}-helix is in blue, turn is in orange and bend is in dark yellow. In (**A-C**) the numbering of residue in each Trx protein is arbitrary and started from the first amino acid in a model. (**D**) The mean conformation of each protein, calculated for MD trajectory **1**, is superimposed on its experimentally determined structures of ERP18, PDI and Tmx1 (in grey), and on the homology model of Tmx4. MD conformations of each protein are shown as colored ribbons – ERp18 in yellow, PDI in red, Tmx1 in green and Tmx4 in blue – with cysteine residue as yellow balls.

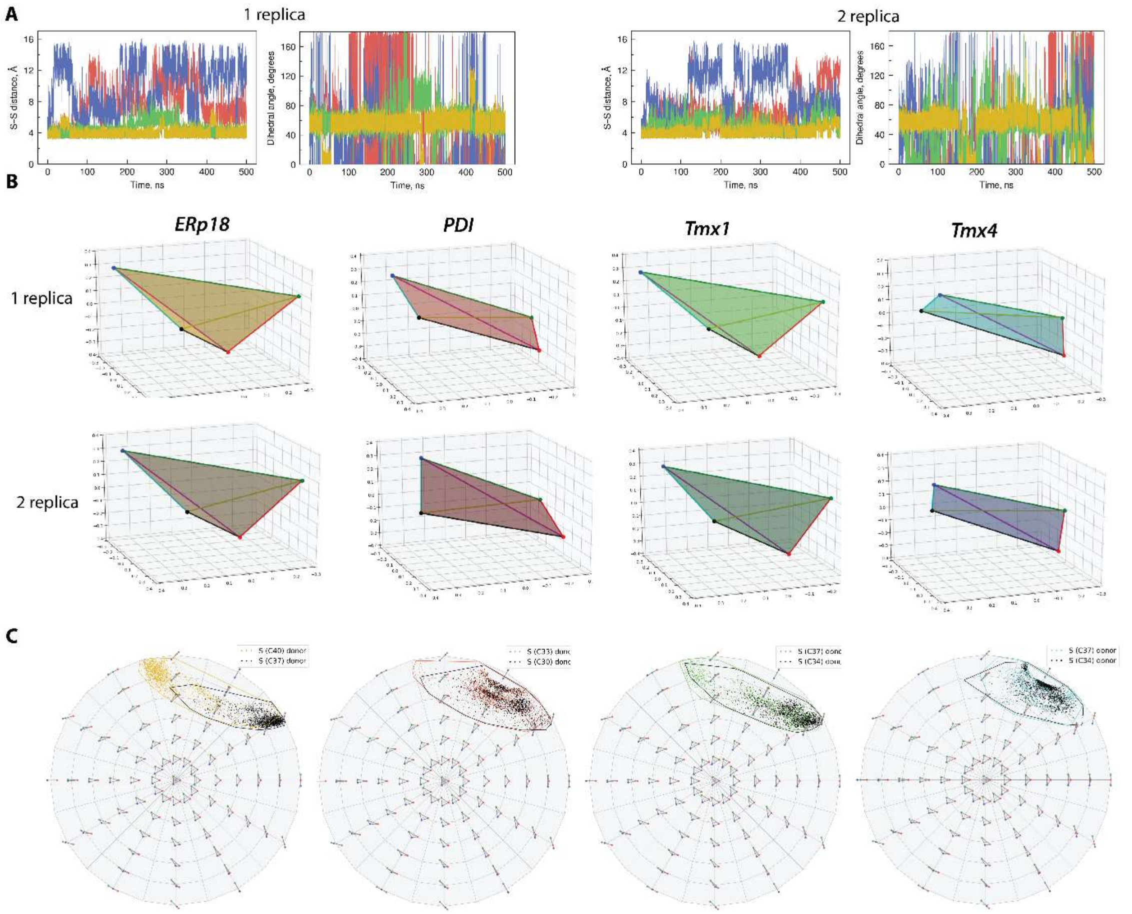

Figure S3. Geometry of CX_1X_2C motif for ERp18, PDI, Tmx1 and Tmx4. (**A**) Geometry of CX_1X_2C motif in each replica is described by distance S···S′ (left) and dihedral angle (right) determined as an absolute value of the pseudo torsion angle S−Cα(C37)−Cα′(C40)−S′. (**B**) Frechet mean of each replica, computed in Kendall framework. (**C**) Projection of S donor (red) - H(green)···S(blue) triangles on a planar disk, where the S-donor is alternatively on the first (black) or second carbon (color). (**A**-**C**) Proteins are distinguished by colour − ERp18 (yellow), PDI (red), Tmx1 (green) and Tmx4 (blue).

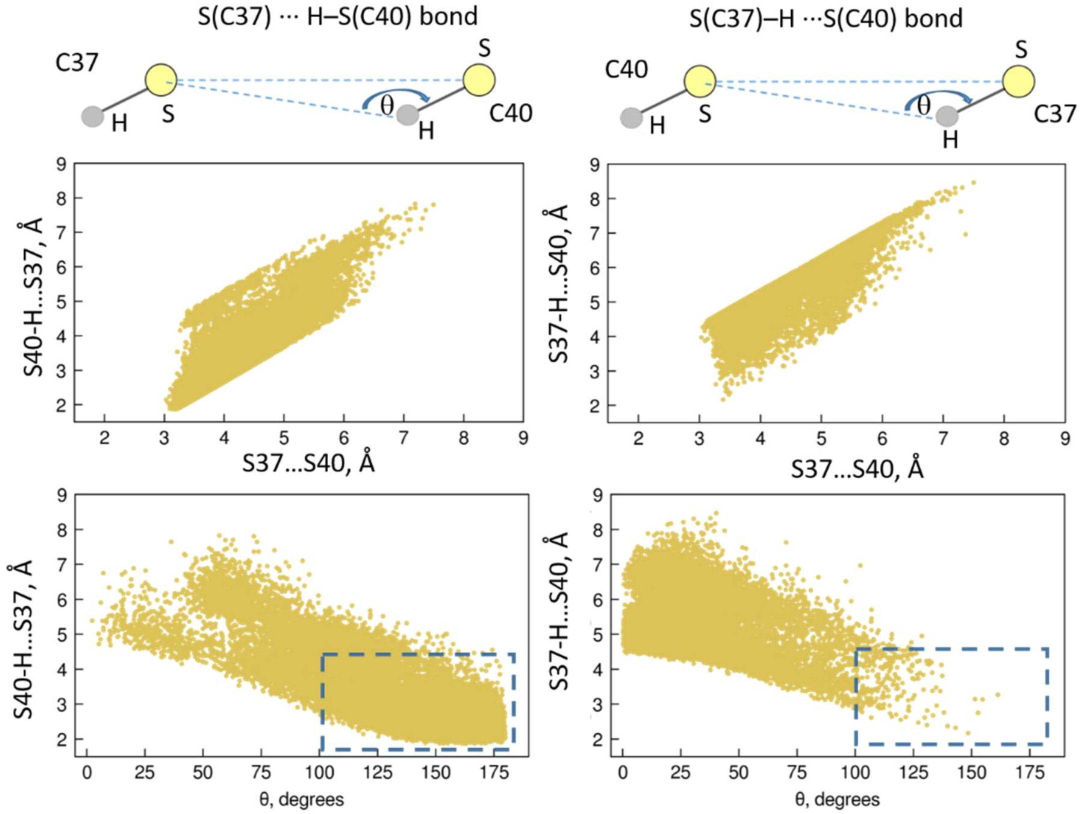

Figure S4. Hydrogen bond in CX_1X_2C fragment from ERp18. Two thiol groups from C40 and C37 are associated by H-bond in which the sulphur atoms from each cysteine residue are the donor (C40) and acceptor (C37) groups respectively. The H-bond is characterised by the mutually correlated parameters, the interatomic distances S···S and the pseudo-covalent angle at H-atom (SH···S). The regions delimited by dashed lines correspond to the H-bond interaction.

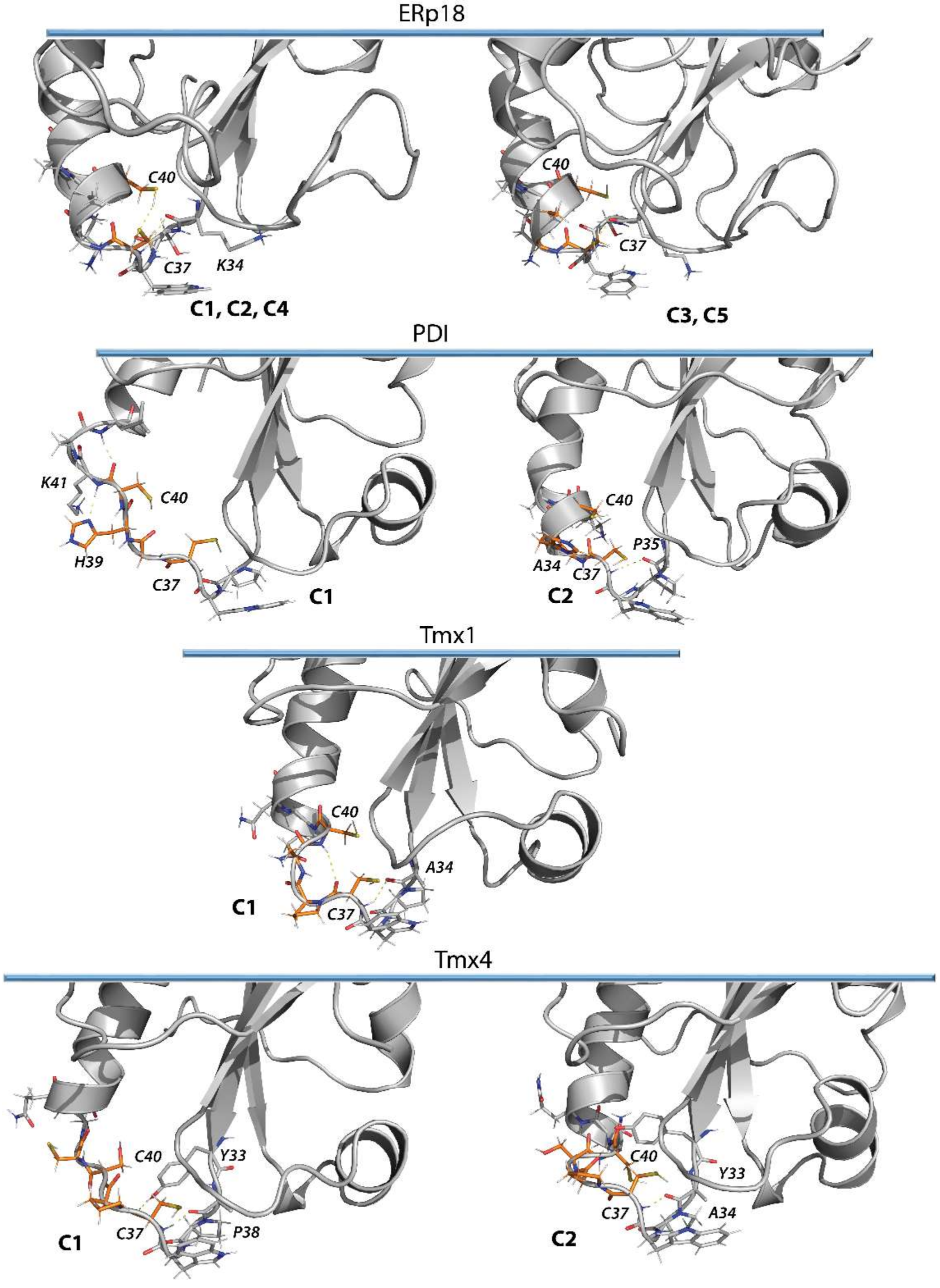

Figure S5. The intra-molecular interactions in the CX_1X_2C motif with neighbour residues was characterised for the conformations regrouped into clusters (cut-off of 2.0 Å) from the concatenated MD trajectory. To regroup the most similar MD conformations and to measure the structural differences between them, after removing the residues with the largest fluctuations from the N- and C-terminals (if its needed), ensemble-based clustering [8] was applied to the concatenated trajectory of each protein. Using the same cut-off value (2.0 Å), results in only one unique cluster encompassing 99% of the MD conformations for Tmx1, two clusters with populations of 94 and 4% in Tmx4, three clusters populated with 73, 14 and 12% of the conformations in PDI, and a large number of clusters with lower populations (38, 17, 15, 13, 5 %) in ERp18. The content of each statistically significant cluster was used for analysis of intramolecular interactions that stabilised the CX_1X_2C motif. Proteins are shown as grey ribbons along with the CX_1X_2C motif and neighbouring residues as sticks.

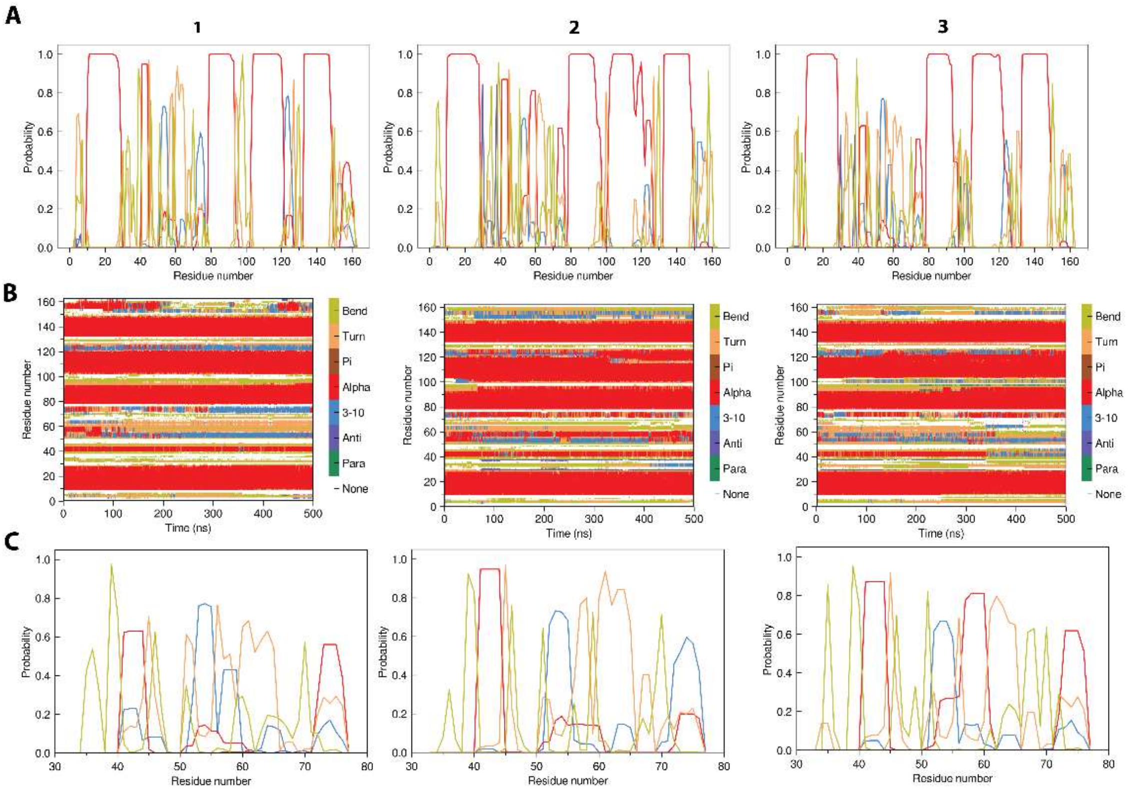

Figure S6. Folding of hVKORC1 over the MD simulations. Secondary structure of each residue of hVKORC1 (**A**) and of L-loop (**C**) assigned by DSSP. Assignment of the secondary structures to colors is given as follows: α-helix is in red, 3_{10}-helix is in blue, the parallel and antiparallel strands are in green and violet respectively; turn is in orange and bend is in dark yellow. Proportion of every secondary structure type for each residue is given as a probability. (**B**) The time-related evolution of the secondary structures of each residue as assigned by DSSP with the type-coded secondary structure bar.

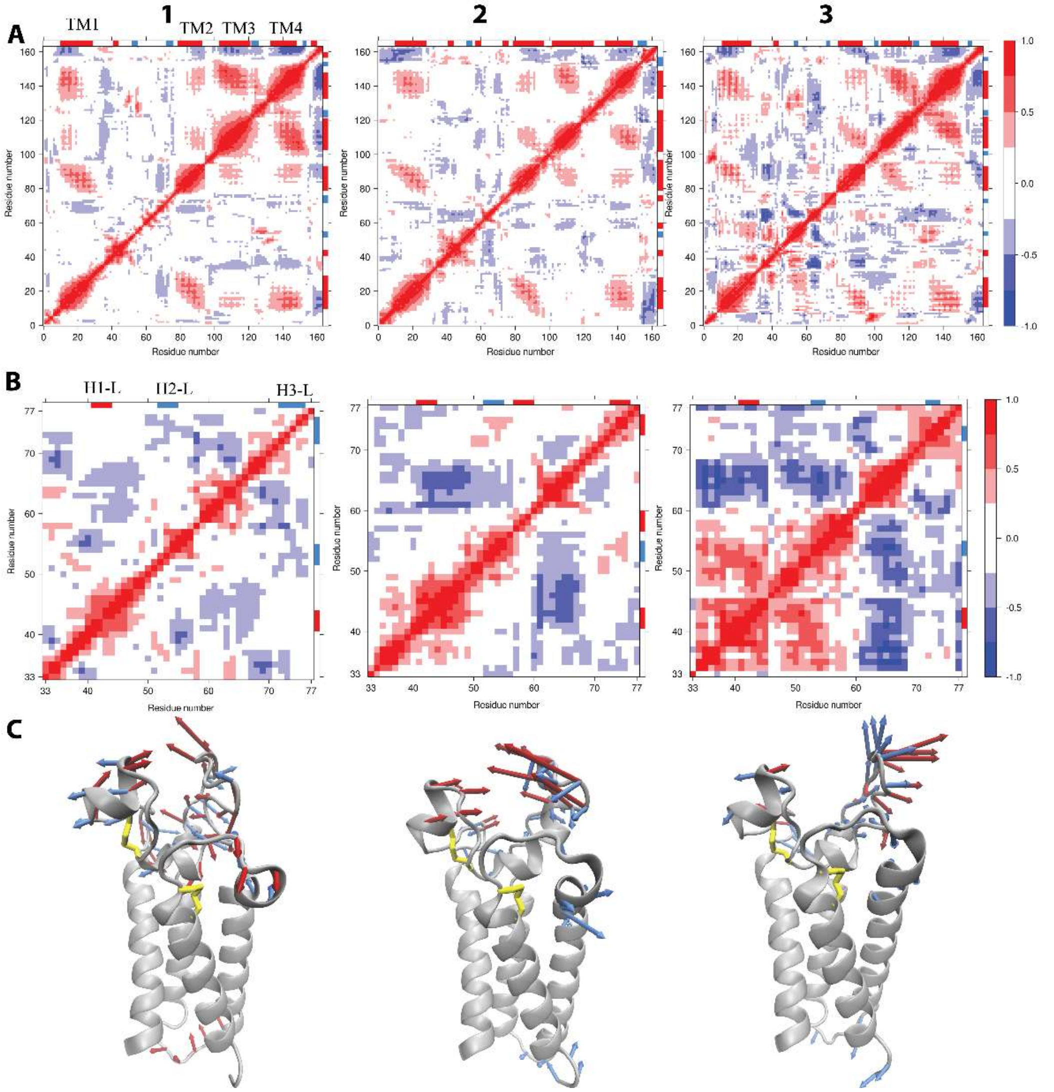

Figure S7. Intrinsic motion of hVKORC1 and its L-loop. Inter-residue cross-correlation map computed for the Cα-atom pairs after fitting on the respective first conformation (t=0ns) of the full-length hVKORC1 (**A**) and of the L-loop (**B**) over each replica **1**-**3**. Correlated (positive) and anti-correlated (negative) motion between the Cα-atom pairs are shown as a red-blue gradient. (**B**) Atomic components in the first PCA modes of hVKORC1 (after omitting the highly fluctuated residues from the N- and C-terminals) are drawn as red (1st mode) and blue (2nd mode) arrows projected onto the respective average structure. Only the motion with an amplitude ≥ of 2 Å was represented. The protein is shown as ribbons diagrams with the S-S bridge as yellow sticks. All computation was performed on the Cα-atoms with the RMSF fluctuations less than 4 Å of each protein after fitting on initial conformation.

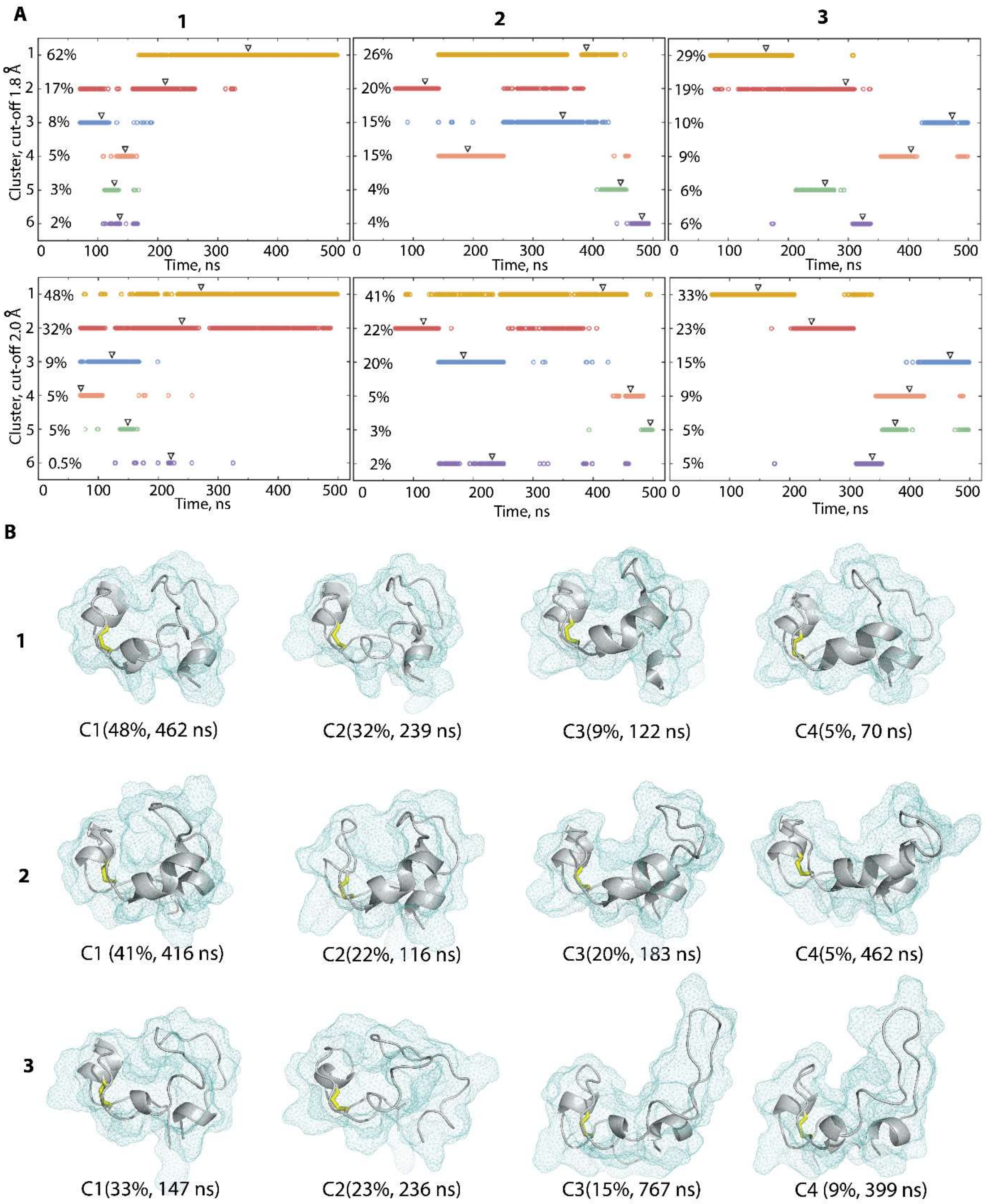

Figure S8. Clustering analysis of the L-loop conformations by using the ensemble-based clustering. (**A**) Regrouping of clusters over each trajectory of MD simulation. Calculation was performed on every 10-ps frames after omitting of the first 70 ns with using the cut-off values of 1.8 (top panel) and 2.0 Å (bottom panel). Clusters classified from the most to the less populated (C1-C6) and affiliated to the time of MD trajectory. Triangle symbol indicated the frames used as the *representative conformations*. (**B**) The *representative conformation* of the L-loop (shown as ribbon with a meshed surface and the S-S bridge as sticks) from each cluster with population >4% (cut-off of 2.0 Å). Population of each cluster is given in brackets together with the time of observation of the *representative conformation*.

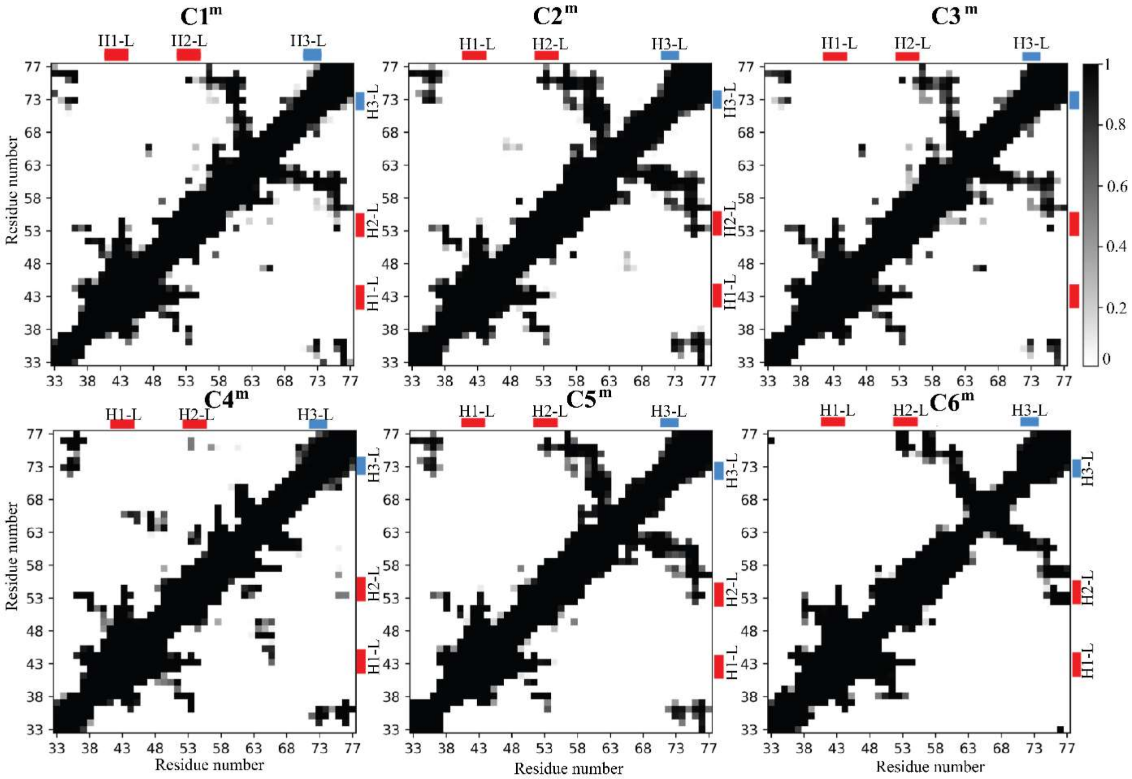

Figure S9. The contact maps of pairwise distances Cα−Cα (< 10 Å) computed for each conformation from the mostly populated clusters (> 4%) found on the concatenated trajectory. Gradient from white (0) to black (1) shows a frequency of the contact during the simulation.

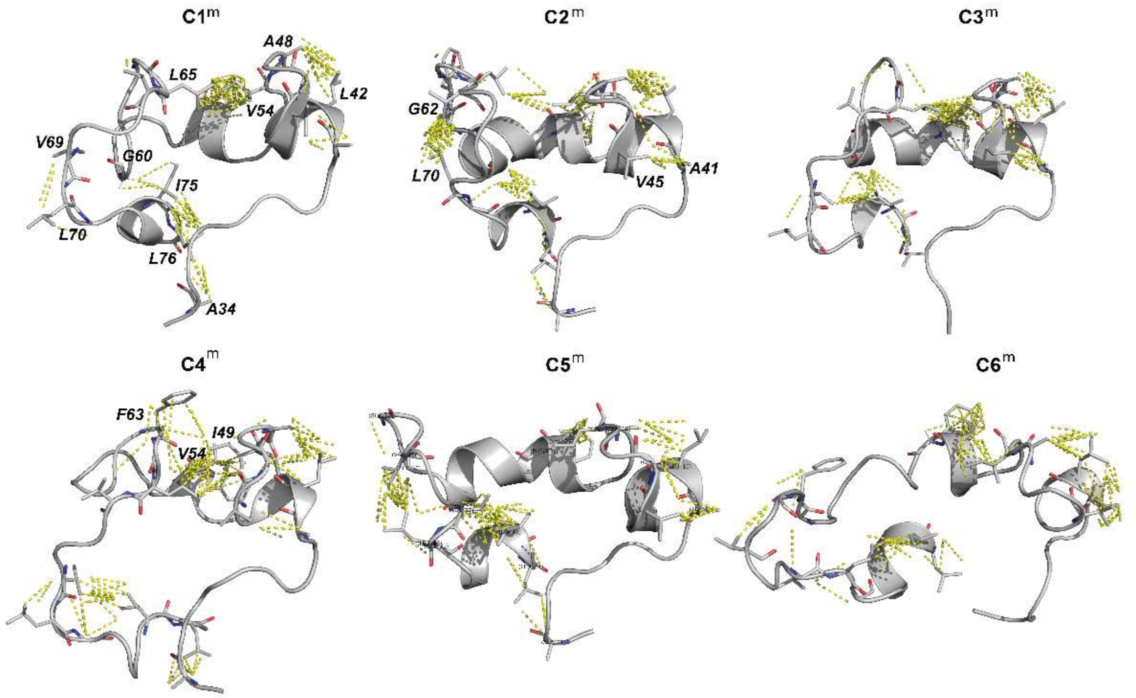

Figure S10. **The hydrophobic contacts (yellow dashed lines) in L-loop of hVKORC1.** The labels of residues are shown on conformation from the cluster C1m; the other labels were added if are required.

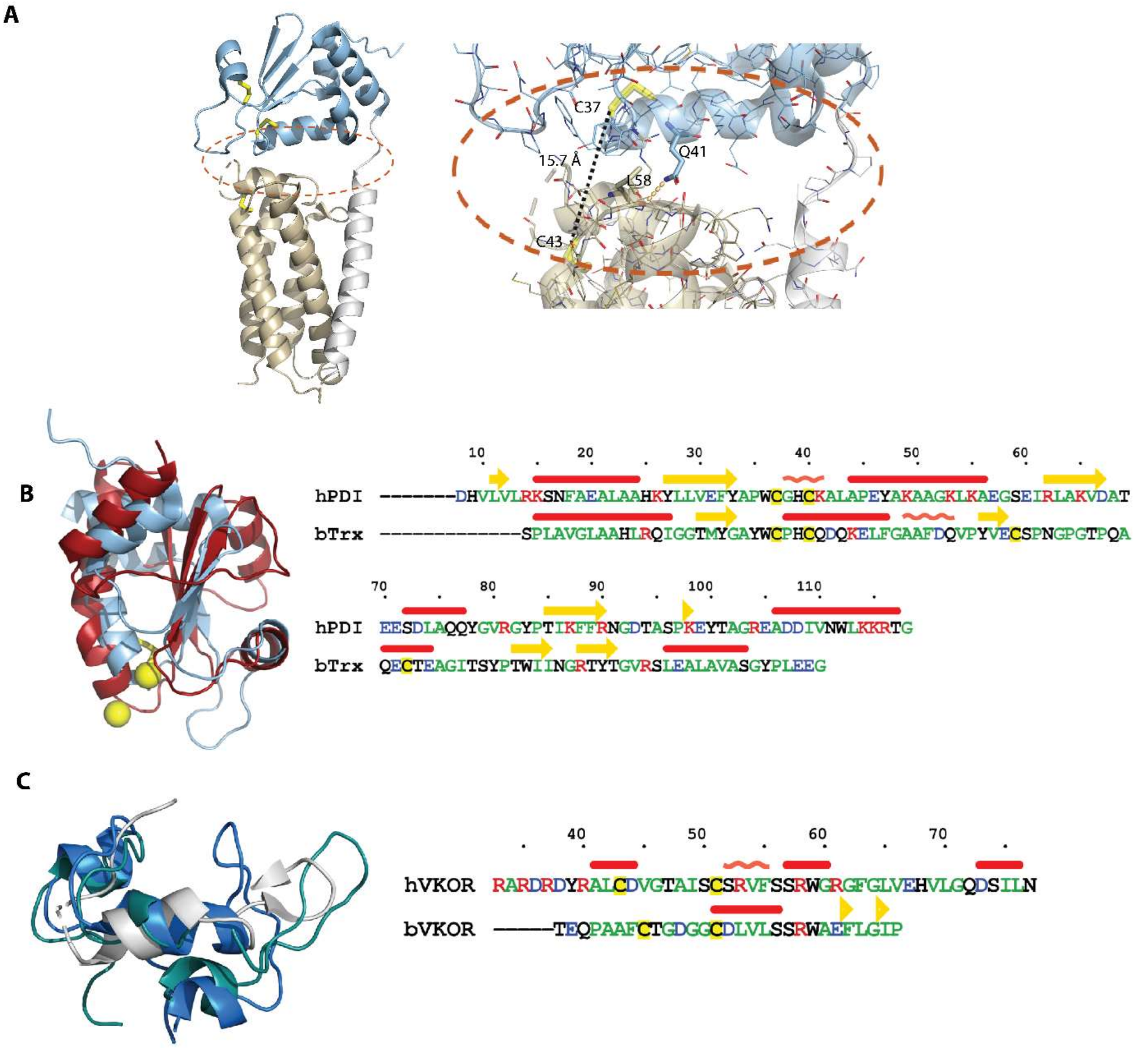

Figure S11. Comparison of the Trx-domain and L-loop from bVKOR with the PDI and L-loop from hVKORC1. (**A**) Structure of bVKOR (PDB ID: 4NV5) and a zooming on the interface region (encircled). Distance between the sulphur atoms from C37 (Trx) and from C43 (L-loop) and the H-bond contact between two domains are shown as dashed lines. (**B**) Superposition of the Trx-like domain from bVKOR (blue) and of the human PDI (red) (RMSD value of 6 Å) (left). The pairwise alignment (NEEDLE program) of sequences of the Trx-like domain from bVKOR and of the human PDI (identity/similarity of 15/20%) (right). (**C**) Superposition of the L-loop from bVKOR (grey) and from hVKORC1 with the 'closed' (blue) and 'open' (cyan) conformations. RMSD values between the L-loop from bVROR and from hVKORC1 are 4.5 and 4 Å for the 'closed' and 'open' conformations (left). The pairwise alignment (NEEDLE program) of the L-loop sequences from bVKOR and from the human PDI (identity and similarity values of 15/20% (right).

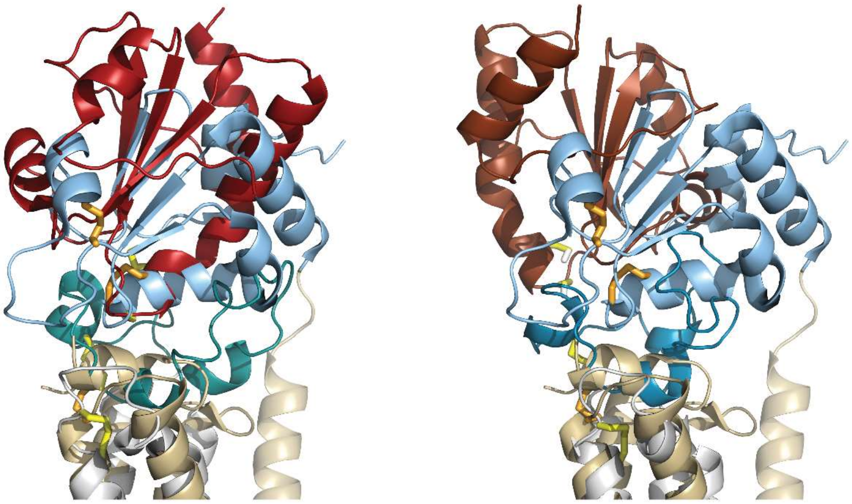

Figure S12. Superposition of **Model 1** (left) and of **Model 2** (right) of the PDI-hVKORC1 complex into the structure of bVKOR (PDB ID: 4NV5). **Model 1** and **Model 2** are represented by conformations taken at t = 80 ns of the stepped finite-time MD simulations. Proteins are shown as coloured ribbons. In the models: PDI in red (**Model 1**) and in brown (**Model 2**), hVKORC1 in grey with L-loop in cyan and blue. In the X-ray structure of bVKORC1: The Trx-like and VKOR-like domains are shown in light blue and in beige respectively. The cysteine residues are shown as sticks, orange in bVKOR and yellow in Models.

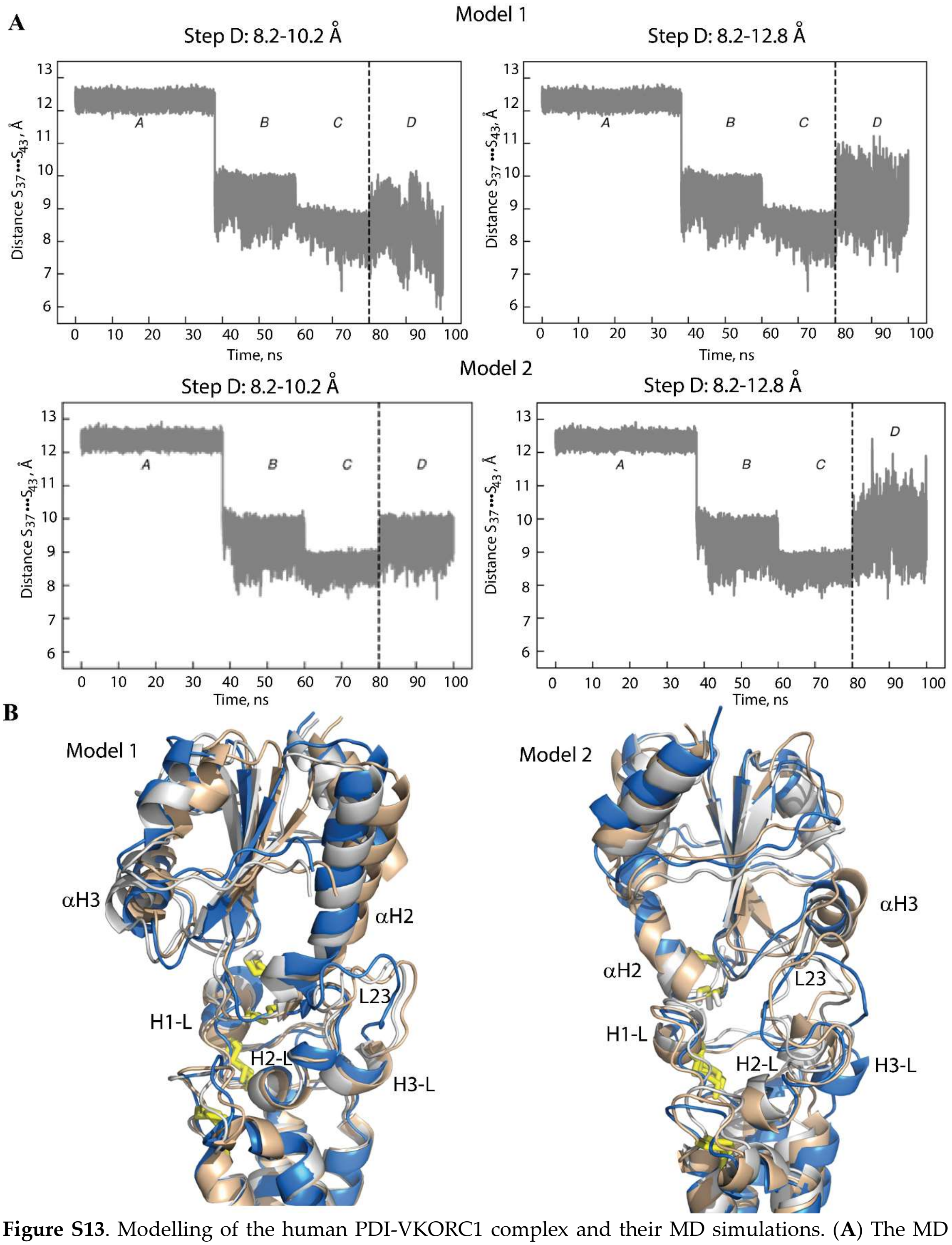

Figure S13. Modelling of the human PDI-VKORC1 complex and their MD simulations. (**A**) The MD simulations of 3D models PDI-VKORC1 complexes, **Model 1** and **Model 2**, were performed with a gradually diminished distance (from 12 to 8 Å) (steps A-C) between the sulphur (S) atoms of C37 from PDI and of C43 from L-loop of hVKORC1, and further (step D) this distance was enlarged to 8.2-12.2 Å and to 8.2-12.8 Å. (**B**) Superimposition of confirmations of Model 1 (left) and Model 2 (right) picked at t=80 (grey) and at 100 ns with S···S distance of 8.2-10.2 Å (beige) and of 8.2-12.8 Å (blue). Proteins are depicted as ribbons. The reference fragments are labelled.

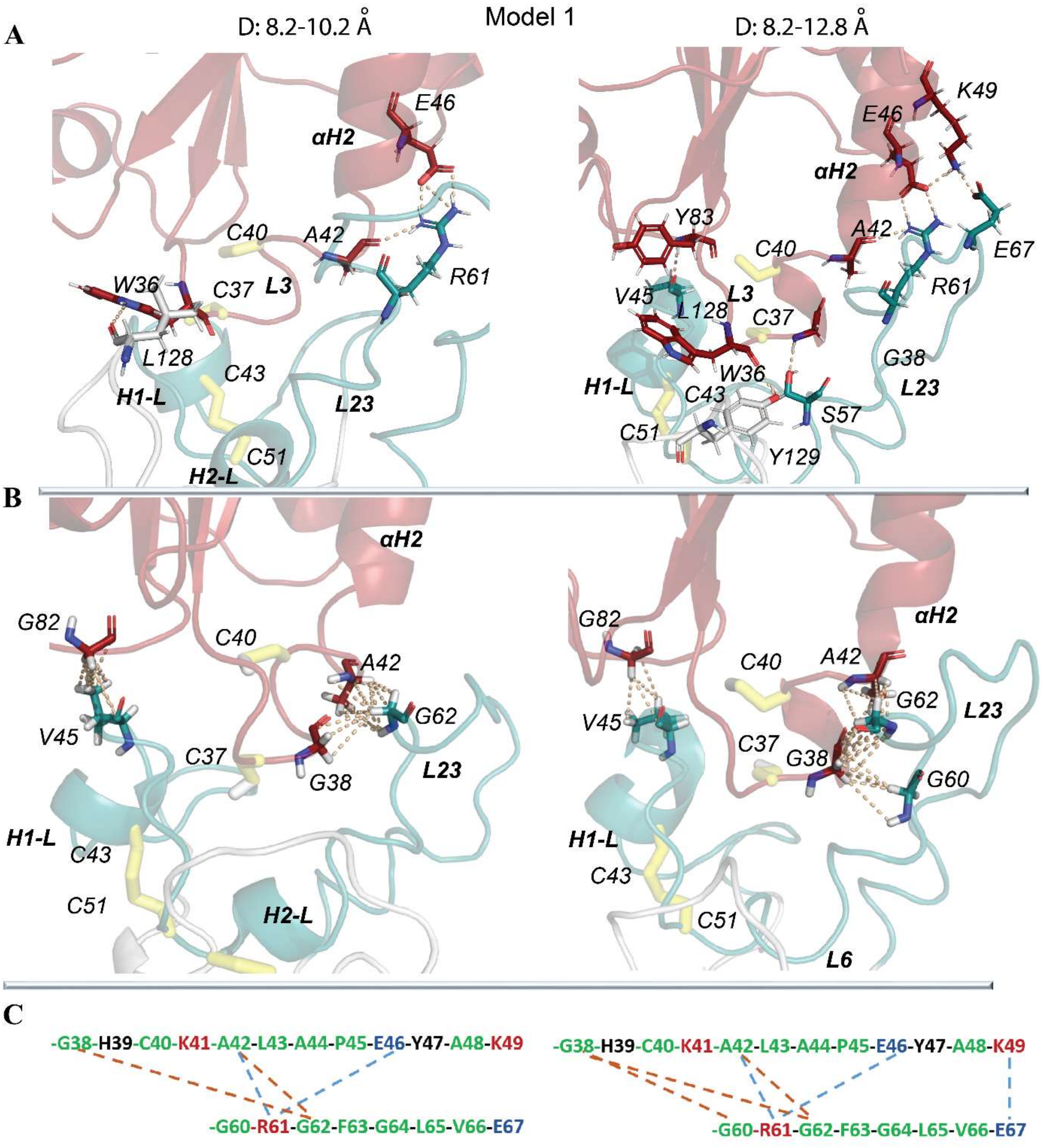

Figure S13. Intermolecular contacts at the interface between PDI and hVKORC1 in the Model 1 of the PDI-hVKORC1 complex. (**A**) The intermolecular H-bonds and (**B**) hydrophobic contacts between PDI and VKORC1, observed in conformations generated using different 'soft' constrains. The proteins a shown as coloured ribbons, PDI in red and brown, and VKORC1 in cyan (L-loop) with the interacting residues and thiol groups as sticks. The contacts are indicated by dashed lines, H-bonds in yellow and hydrophobic in salmon. The structural fragments and residues participating in the contacts are labelled. Analysis of intermolecular contacts was performed on conformations taken at t=80 ns. (**C**) A pattern of H-bond (in blue) and hydrophobic (in orange) contacts between the PDI and hVKORC1 residues. Residues are coloured according to their properties – the positively and negatively charged residues are in red and blue respectively, the hydrophobic residues are in green, the polar and amphipathic residues are in black.

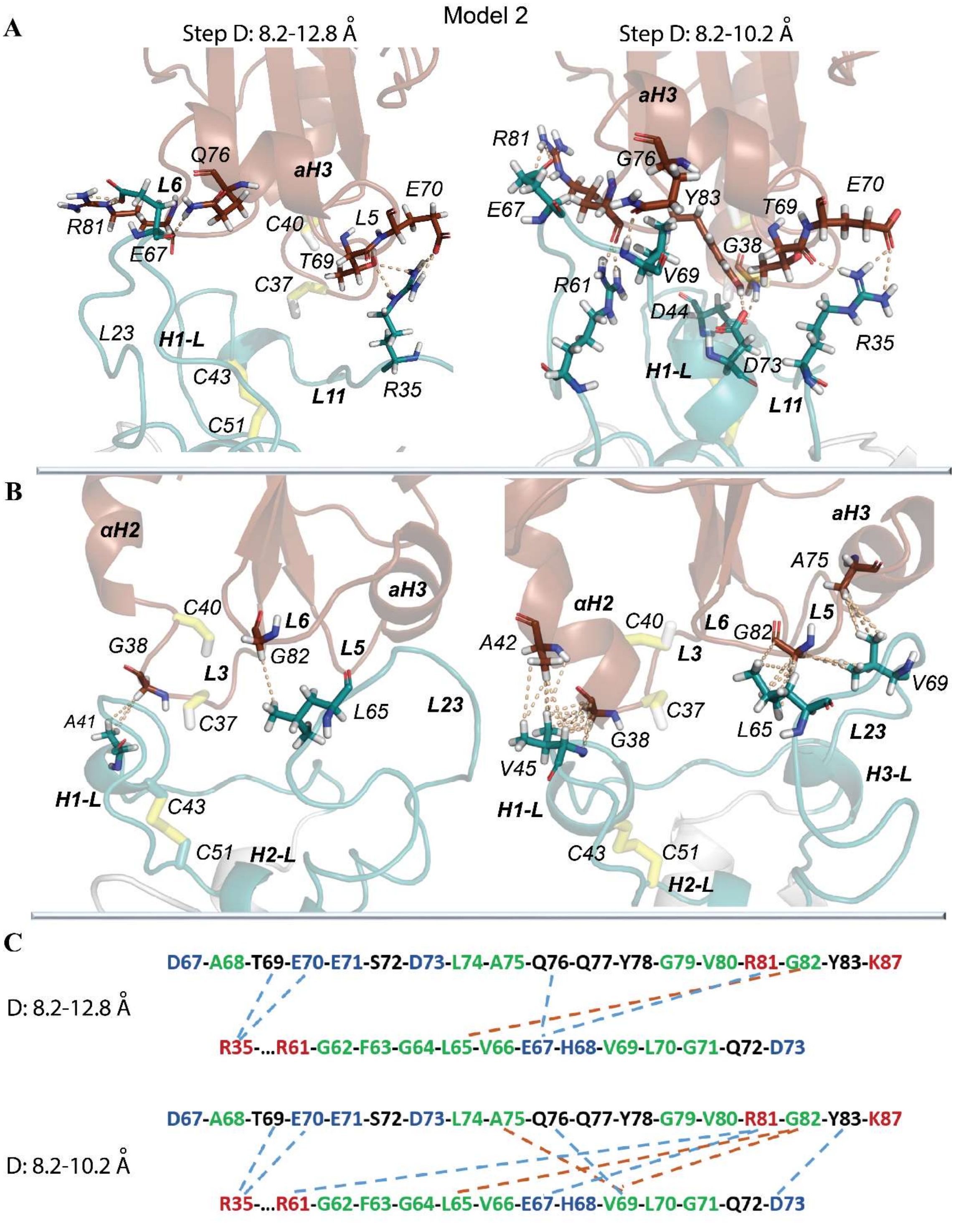

Figure A14. Intermolecular contacts at the interface between PDI and hVKORC1 in the Model 2 of the PDI-hVKORC1 complex. (**A**) The intermolecular H-bonds and (**B**) hydrophobic contacts between PDI and VKORC1, observed in conformations generated using different 'soft' constrains. The proteins a shown as coloured ribbons, PDI in red and brown, and VKORC1 in cyan (L-loop) with the interacting residues and thiol groups as sticks. The contacts are indicated by dashed lines, H-bonds in yellow and hydrophobic in salmon. The structural fragments and residues participating in the contacts are labelled. Analysis of

intermolecular contacts was performed on conformations taken at t=80 ns. (**C**) A pattern of H-bond (in blue) and hydrophobic (in orange) contacts between the PDI and hVKORC1 residues. Residues are coloured according to their properties – the positively and negatively charged residues are in red and blue respectively, the hydrophobic residues are in green, the polar and amphipathic residues are in black.

References

1. Berman HM, Westbrook J, Feng Z, Gilliland G, Bhat TN, Weissig H, et al. The Protein Data Bank. Nucleic acids research. 2000;28(1):235-42. Epub 1999/12/11. doi: 10.1093/nar/28.1.235. PubMed PMID: 10592235; PubMed Central PMCID: PMCPMC102472.
2. Kemmink J, Darby NJ, Dijkstra K, Nilges M, Creighton TE. Structure determination of the N-terminal thioredoxin-like domain of protein disulfide isomerase using multidimensional heteronuclear 13C/15N NMR spectroscopy. Biochemistry. 1996;35(24):7684-91. Epub 1996/06/18. doi: 10.1021/bi960335m. PubMed PMID: 8672469.
3. Wang C, Yu J, Huo L, Wang L, Feng W, Wang C-c. Human protein-disulfide isomerase is a redox-regulated chaperone activated by oxidation of domain a'. The Journal of biological chemistry. 2012;287(2):1139-49. Epub 11/16. doi: 10.1074/jbc.M111.303149. PubMed PMID: 22090031.
4. Wang L, Zhang L, Niu Y, Sitia R, Wang CC. Glutathione peroxidase 7 utilizes hydrogen peroxide generated by Ero1α to promote oxidative protein folding. Antioxidants & redox signaling. 2014;20(4):545-56. Epub 2013/08/08. doi: 10.1089/ars.2013.5236. PubMed PMID: 23919619; PubMed Central PMCID: PMCPMC3901321.
5. Biterova EI, Isupov MN, Keegan RM, Lebedev AA, Sohail AA, Liaqat I, et al. The crystal structure of human microsomal triglyceride transfer protein. Proceedings of the National Academy of Sciences. 2019;116(35):17251-60. doi: 10.1073/pnas.1903029116.
6. Rowe ML, Ruddock LW, Kelly G, Schmidt JM, Williamson RA, Howard MJ. Solution Structure and Dynamics of ERp18, a Small Endoplasmic Reticulum Resident Oxidoreductase. Biochemistry. 2009;48(21):4596-606. doi: 10.1021/bi9003342.
7. Madeira F, Park Ym, Lee J, Buso N, Gur T, Madhusoodanan N, et al. The EMBL-EBI search and sequence analysis tools APIs in 2019. Nucleic acids research. 2019;47(W1):W636-W41. doi: 10.1093/nar/gkz268.
8. Lyman E, Zuckerman DM. Ensemble-based convergence analysis of biomolecular trajectories. Biophysical journal. 2006;91(1):164-72. Epub 2006/04/18. doi: 10.1529/biophysj.106.082941. PubMed PMID: 16617086; PubMed Central PMCID: PMCPMC1479051.
